# Sodium Butyrate Attenuates Isoprenaline-Induced Myocardial Injury via Restoring the Gut–Heart Axis and Suppressing TLR4/NF-κB Signaling

**DOI:** 10.3390/cimb48050501

**Published:** 2026-05-13

**Authors:** Hazrat Bilal, Imran Khan, Ayesha Yaseen, Xiaopeng Zhang, Xuexue Liu, Jian Zhao, Jing Li, Ata Ur Rehman, Lei Sun, Xiao Yu

**Affiliations:** 1 Department of Pathology and Forensic Medicine, College of Basic Medical Sciences, Dalian Medical University, Dalian 116044, China; hb99977@hotmail.com (H.B.); aishamalik.aigs@yahoo.com (A.Y.); 18304097427@163.com (X.Z.); 18735829575@163.com (X.L.); zhaojian_19940130@163.com (J.Z.); a15898132596@163.com (J.L.); 2 Department of Microecology, College of Basic Medical Sciences, Dalian Medical University, Dalian 116044, China; imran.mic727@gmail.com; 3 Department of Pharmacology, University of Virginia, Charlottesville, VA 22908, USA; dratadmu1947@gmail.com

**Keywords:** isoprenaline, myocardial injury, sodium butyrate, gut microbiota, gut–heart axis, TLR4/NF-κB signaling, metabolomics

## Abstract

The gut–heart axis plays a role in cardiac injury due to the disruption of barriers, endotoxemia, and inflammatory signaling; yet, it is not clear whether sodium butyrate (SB) is capable of alleviating isoprenaline-induced myocardial injury through coordinated intestinal, microbial, and metabolic restoration. This study used male Sprague-Dawley rats, which were grouped into control, control + SB, isoprenaline (ISO)-induced myocardial injury, and ISO + SB groups. We evaluated cardiac biomarkers of injury, oxidative stress, histopathologic, intestinal barrier (16S rRNA sequencing), and serum metabolomics (LC-MS). SB treatment decreased serum cardiac troponin I, creatine kinase-MB, and lactate dehydrogenase; relieved oxidative stress; and lowered myocardial necrosis and fibrosis. It re-established colonic architecture, upregulated the expression of ZO-1 (zonula occludens-1) and claudin-1, and reduced endotoxin in the bloodstream. SB also prevented the production of proinflammatory cytokines such as TNF-α, IL-6, and IL-1β; cardiac TLR4; IκBα degradation; and NF-κB p 65 phosphorylation. In addition, SB altered the gut microbiota in favor of beneficial commensals, including *Ligilactobacillus* and *Bifidobacterium*, and reduced *Desulfovibrio*. It normalized key circulating metabolites and enriched cardiometabolic pathways, and the patterns of correlation suggested the coordinated remodeling of the microbiome–metabolome. These findings reveal that SB prevents myocardial injury caused by ISO through strengthening gut barrier protection, alleviating endotoxemia, inhibiting TLR4/NF-κB, and remodeling the microbiome–metabolome axis, indicating its potential for use as a gut-targeted cardioprotective intervention.

## 1. Introduction

Ischemic heart disease, particularly acute myocardial infarction (MI), remains the leading cause of mortality worldwide [1,2]. The pathophysiology of MI extends beyond the initial ischemic event, involving persistent inflammation, oxidative stress, and maladaptive remodeling, which collectively drive progression toward heart failure [3,4,5]. Despite significant advances in reperfusion strategies and pharmacotherapy, substantial residual inflammatory risk persists, underscoring the need for therapeutic approaches that target the systemic drivers of post-infarction pathology [6,7].

The intestinal microbiota is increasingly recognized as a metabolically active organ that profoundly influences cardiovascular homeostasis through the gut–heart axis [8,9]. This bidirectional communication network involves microbial metabolites, neuroendocrine signaling, and the modulation of host immunity [10,11,12]. Microbial dysbiosis has been implicated in hypertension, atherosclerosis, and heart failure, where altered microbial composition promotes systemic inflammation and metabolic dysfunction [9].

Acute MI triggers sympathetic activation, intestinal hypoperfusion, and inflammatory stress, which compromise epithelial barrier integrity and increase intestinal permeability [13,14]. The translocation of bacterial lipopolysaccharide (LPS) into the circulation activates pattern recognition receptors, including Toll-like receptor-4 (TLR4), leading to nuclear factor kappa light chain enhancer of activated B cells (NF-κB)-mediated inflammatory signaling [13,15]. This gut-derived inflammatory cascade contributes to myocardial injury and adverse remodeling following ischemic damage [16].

Short-chain fatty acids (SCFAs), particularly acetate, propionate, and butyrate, are key functional metabolites generated by the microbial fermentation of dietary fiber [17,18]. Butyrate serves as the principal energy substrate for colonocytes and reinforces epithelial barrier integrity by promoting tight junction expression [19]. Beyond local intestinal effects, butyrate exerts systemic anti-inflammatory and immunomodulatory actions, partly through the inhibition histone deacetylase (HDAC) and the regulation of immune cell differentiation [20,21]. Emerging evidence suggests SCFAs influence cardiovascular inflammation and remodeling through NF-κB-related signaling pathways [22].

Although dietary or probiotic strategies can enhance endogenous SCFA production and confer cardiovascular benefits in experimental models [23,24], direct pharmacological supplementation with sodium butyrate provides a more controlled approach for mechanistic evaluation. However, whether sodium butyrate simultaneously preserves intestinal barrier integrity, modulates gut microbiota composition, and restores systemic metabolic homeostasis after myocardial injury remains insufficiently defined. Therefore, this study employed an integrated multi-omics strategy combining histopathology, immunofluorescence, 16S rRNA sequencing, and untargeted metabolomics in an isoprenaline-induced rat model of myocardial injury to test whether oral sodium butyrate confers cardioprotection through coordinated gut–heart axis restoration. The specific novelty lies in mechanistically linking gut barrier disruption to cardiac TLR4/NF-κB activation, simultaneously characterizing microbial and metabolomic reprogramming, and identifying functional host–microbe interactions mediating cardioprotection.

## 2. Materials and Methods

### 2.1. Reagents

Isoprenaline hydrochloride (Cat# T1056) was obtained from TargetMol (Boston, MA, USA) and sodium butyrate (Cat# 303410) from Sigma Aldrich (St. Louis, MO, USA). ELISA kits for cardiac troponin I (cTnI; Cat# SYP-R0289; Upingbio, Shanghai, China), tumor necrosis factor-alpha (TNF-α; Cat# CSB-E11987r), interleukin-6 (IL-6; Cat# CSB-E04640r), and interleukin-1β (IL-1β; Cat# CSB-E08055r) were purchased from Cusabio (Wuhan, China). An endotoxin (LPS) assay kit (Cat# BPE30092) was obtained from Lengton (Shanghai, China). Assay kits for creatine kinase-MB (CK-MB), lactate dehydrogenase (LDH), malondialdehyde (MDA), and superoxide dismutase (SOD) were supplied by Nanjing Jiancheng Bioengineering Institute (Nanjing, China). Masson’s trichrome stain kit (Cat# G1340) was purchased from Solarbio (Beijing, China). The cDNA synthesis kit (Cat# SM135) and SYBR Green Master Mix (Cat# SM143) were obtained from Sevenbio (Beijing, China). Primary antibodies included anti-TLR4 (19811-1-AP; 1:1000), anti-IκBα (66418-1-Ig; 1:1000) from Proteintech (Wuhan, China) and phospho-NF-κB p65 (CST #3033; 1:1000) from Cell Signaling Technology (Danvers, MA, USA). HRP-conjugated anti-β-actin (Cat# ZB15001-HRP; 1:5000) was purchased from Servicebio (Wuhan, China). Anti-Claudin-1 (bs-1428R) and anti-ZO-1/*TJP1* (bs-1329R) were obtained from Bioss (Beijing, China). Secondary antibodies were purchased from Proteintech (Wuhan, China).

### 2.2. Animal Model and Experimental Design

Male Sprague-Dawley (SD) rats, weighing 250–300 g, were obtained from the Institutional Animal Facility of Dalian Medical University. Animals were housed under standard laboratory conditions (22 ± 2 °C, 55 ± 5% relative humidity, 12 h light/dark cycle) with ad libitum access to standard chow and water. All experimental procedures were approved by the Animal Ethics Committee of Dalian Medical University (Approval Number: 202310280) and performed in accordance with institutional guidelines for the care and use of laboratory animals. Each experimental group comprised 6 male Sprague-Dawley rats (*n* = 6 per group; 24 animals total). Following one week of acclimatization, rats were randomly assigned to four groups: 1. control (CON): received subcutaneous (s.c.) injections of sterile saline for two consecutive days; 2. control + sodium butyrate (CON-SB): received s.c. saline for two days, followed by oral sodium butyrate administration (300 mg/kg/day) for 4 weeks; 3. ISO-induced myocardial injury model (MOD): received s.c. injections of isoprenaline (ISO; 85 mg/kg) for two consecutive days to induce myocardial injury; 4. ISO + sodium butyrate (MOD-SB): received s.c. ISO (85 mg/kg) for two consecutive days, followed by oral sodium butyrate administration (300 mg/kg/day) for 4 weeks. The dosages of ISO and sodium butyrate were selected based on previously published studies [25,26,27]. At the end of the treatment period, the rats were anesthetized with pentobarbital sodium, and blood, heart, and colon tissues were harvested for subsequent biochemical, histological, and molecular analyses.

### 2.3. Biochemical and Inflammatory Marker Analysis

Serum cardiac troponin I (cTnI), creatine kinase-MB (CK-MB), and lactate dehydrogenase (LDH) levels were measured using commercially available kits according to the manufacturer’s instructions. All biochemical assays were performed on individual biological samples (*n* = 6 per group) in technical duplicates, and results were averaged for statistical analysis. Serum levels of tumor necrosis factor-alpha (TNF-α), interleukin-6 (IL-6), and interleukin-1β (IL-1β) were quantified by ELISA. Left ventricular tissue was homogenized in ice-cold buffer and centrifuged at 3000× *g* for 15 min at 4 °C, and the supernatant was collected. Malondialdehyde (MDA) content and superoxide dismutase (SOD) activity were measured using commercial colorimetric assay kits.

### 2.4. Histopathological Analysis

Heart and colon tissues were fixed in 10% neutral buffered formalin for 48 h, dehydrated through a graded ethanol series, cleared in xylene, and embedded in paraffin. Sections (3 µm) were prepared and stained with hematoxylin and eosin (H&E) for histological assessment and with Masson’s trichrome for fibrosis evaluation. Images were obtained using an Olympus IX73 microscope (Tokyo, Japan).

### 2.5. Western Blot Analysis

Total protein was extracted from cardiac tissue using RIPA lysis buffer supplemented with protease and phosphatase inhibitors. Protein concentrations were determined using a bicinchoninic acid (BCA) assay. Equal amounts of protein were separated by SDS-PAGE and transferred onto nitrocellulose membranes. Membranes were blocked with 5% non-fat milk for 1 h at room temperature and incubated overnight at 4 °C with primary antibodies against TLR4, IκBα, and phosphorylated NF-κB p65. Following washing, membranes were incubated with appropriate HRP-conjugated secondary antibodies for 1 h at room temperature. Protein bands were visualized using enhanced chemiluminescence (ECL) and quantified by densitometry using ImageJ software (version 1.53, National Institutes of Health, Bethesda, MD, USA). Quantification was performed on a per lane basis: each target protein band was individually normalized to the β-actin band from the same lane.

### 2.6. Quantitative Real-Time PCR (qRT-PCR)

Total RNA was isolated from colon tissue using TRIzol reagent (Thermo Fisher Scientific, Waltham, MA, USA) according to the manufacturer’s protocol. RNA concentration and purity were evaluated using a NanoDrop spectrophotometer (Thermo Fisher Scientific, Waltham, MA, USA). One microgram of total RNA was reverse transcribed into complementary DNA (cDNA) using the All-in-one First Strand cDNA Synthesis Kit III (Vazyme, Nanjing, China). Quantitative real-time PCR was performed in triplicate on a LineGene 9600 system (Bioer, Hangzhou, China) using SYBR Green PCR Master Mix. Relative mRNA expression levels of tight junction genes *Tjp1* (ZO-1) and *Cldn1* (claudin-1) were calculated using the 2^−ΔΔCT^ method with *Actb* (β-actin) as the internal reference gene. Primer sequences are provided in Table 1.

### 2.7. Immunofluorescent Staining

Paraffin-embedded colon sections (5 μm) were baked at 60 °C for 1 h, deparaffinized in xylene (2 × 10 min), and rehydrated through a graded ethanol series. Antigen retrieval was performed in 10 mM sodium citrate buffer (pH 6.0) containing 0.05% Tween-20 at 95 °C for 15 min. Sections were cooled to room temperature and rinsed with phosphate-buffered saline (PBS). To reduce nonspecific binding, sections were blocked with blocking solution for 20 min at room temperature and subsequently incubated overnight at 4 °C with rabbit polyclonal primary antibodies against ZO-1 and claudin-1 (Bioss, China). After PBS washing, slides were incubated for 1 h at room temperature in the dark with CoralLite 488-conjugated goat anti-rabbit IgG secondary antibody. Nuclei were counterstained with 4′,6-diamidino-2-phenylindole (DAPI, 1 μg/mL) for 5 min. Finally, sections were rinsed and mounted with antifade medium, and fluorescence images were captured using a fluorescence microscope. Fluorescence intensity was quantified using ImageJ software.

### 2.8. 16S rRNA Gene Sequencing and Microbiome Analysis

At the end of the experiment, fecal samples were aseptically collected into sterile DNA-free tubes, snap-frozen in liquid nitrogen, and stored at −80 °C. Microbial genomic DNA was extracted using a QIAamp PowerFecal Pro DNA Kit (Qiagen, Hilden, Germany) following the manufacturer’s instructions. The V3–V4 hypervariable region of the bacterial 16S rRNA gene, which provides a balance of broad phylogenetic coverage and sufficient taxonomic resolution for genus-level classification, was amplified using primers 341F (5′-CCTAYGGGRBGCASCAG-3′) and 806R (5′-GGACTACNNGGGTWTCTAAT-3′).

Sequencing libraries were prepared using an Illumina Nextera XT library preparation kit, followed by PCR amplification, purification, and quantification. Purified amplicons were pooled at equimolar concentrations and sequenced on an Illumina MiSeq platform (2 × 300 bp) by Shanghai LingEn Biotechnology (Shanghai, China). Raw reads were demultiplexed, quality-filtered using Trimmomatic (version 0.39), and merged with FLASH (version 1.2.11). Low-quality and chimeric sequences were removed, and operational taxonomic units (OTUs) were clustered at 97% similarity using USEARCH (version 11.0).

Taxonomic classification was performed with the Ribosomal Database Project (RDP) Classifier against the SILVA 138.1 database. After quality control, 31,059–38,843 reads per sample were obtained, generating 2082 OTUs. The OTU table was rarefied to 31,059 reads for diversity analysis. Alpha diversity (observed species, Shannon, Chao1) and beta diversity (unweighted UniFrac) were calculated in QIIME2 (Quantitative Insights Into Microbial Ecology 2), and differential taxa were identified using linear discriminant analysis effect size (LEfSe; α = 0.05, linear discriminant analysis (LDA) score ≥ 2.0).

### 2.9. Untargeted Serum Metabolomics

Serum metabolites were extracted by protein precipitation by mixing 100 µL of serum with 400 µL of 80% methanol, followed by vortexing and centrifuging at 15,000× *g* for 20 min at 4 °C. The supernatant was collected for analysis, and a pooled quality control (QC) sample was prepared by combining equal aliquots from all samples. Untargeted metabolomic profiling was performed using a Vanquish UHPLC system coupled with a Q Exactive HF-X mass spectrometer (Thermo Fisher Scientific, USA), with chromatographic separation on a Hypersil Gold C18 column (Thermo Fisher Scientific, 100 × 2.1 mm, 1.9 µm). Data were acquired in both positive and negative ionization modes over an m/z range of 100–1500. Raw data were processed using XCMS (version 3.12.0; an open-source R package for untargeted metabolomics data processing, run in R version 4.3.1) for peak detection, retention time alignment, and integration. Metabolites were annotated by matching MS/MS spectra against the Human Metabolome Database (HMDB) and the Kyoto Encyclopedia of Genes and Genomes (KEGG) database with a mass tolerance of ±10 ppm. Data preprocessing included blank subtraction and quality control (QC)-based feature filtering (relative standard deviation, RSD, <30%), followed by log_10_ transformation and Pareto scaling. Differential metabolites were identified using orthogonal partial least squares–discriminant analysis (OPLS-DA) conducted in SIMCA-P (version 14.1, Sartorius Stedim Biotech, Umeå, Sweden), with variable importance in projection (VIP) > 1.0 and FDR-adjusted *q* < 0.05 (Benjamini–Hochberg). KEGG pathway enrichment analysis of SB-responsive metabolites was performed using MetaboAnalyst (version 5.0; https://www.metaboanalyst.ca; accessed on 1 September 2025). Metabolites showing opposite directional trends between the MOD vs. CON and MOD-SB vs. MOD comparisons were defined as SB-reversed metabolites.

### 2.10. Statistical Analysis

Data are expressed as mean ± standard error of the mean (SEM). Statistical analyses were performed using GraphPad Prism (version 9.0; GraphPad software, San Diego, CA, USA). For comparisons among experimental groups, one-way ANOVA with Tukey’s post hoc test was applied for normally distributed data. For 16S rRNA gene sequencing and untargeted metabolomics datasets, which typically do not follow a normal distribution, group differences were assessed using the nonparametric Kruskal–Wallis test followed by Dunn’s post hoc test. Spearman’s rank correlation analysis was used to assess associations between gut microbial genera and serum metabolites. Statistical significance was set at *p* < 0.05.

### 2.11. Data Visualization

Volcano plots depicting differential metabolite features were generated in R (version 4.3.1) using the ggplot2 package (version 3.4.2), with significance thresholds of VIP > 1.0 and FDR-adjusted *q* < 0.05. Metabolite heatmaps were produced using the pheatmap package in R with hierarchical clustering (Ward linkage, Euclidean distance) applied to log_10_-transformed, Pareto-scaled data. Venn diagrams summarizing overlaps of differential metabolites were generated using the VennDiagram package in R. Principal component analysis (PCA), principal coordinate analysis (PCoA), and non-metric multidimensional scaling (NMDS) of microbial community data were computed in QIIME2 and visualized using the Emperor plugin and the vegan package in R. The microbiome–metabolome Spearman correlation heatmap was constructed using the corrplot package in R, with the Benjamini–Hochberg FDR correction applied; statistically significant correlations (*q* < 0.05) are indicated by asterisks.

## 3. Results

### 3.1. Sodium Butyrate Alleviated ISO-Induced Myocardial Damage

**Figure 1:**
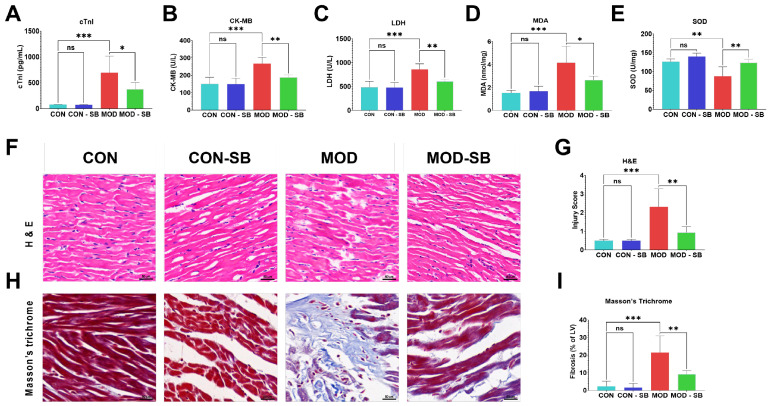
Sodium butyrate attenuated isoprenaline-induced cardiac injury, oxidative stress, and fibrosis. Serum levels of cardiac injury markers (**A**) cardiac troponin (cTnI), (**B**) creatine kinase-MB (CK-MB), and (**C**) lactate dehydrogenase (LDH). Myocardial oxidative stress indicators: (**D**) malondialdehyde (MDA) content and (**E**) superoxide dismutase (SOD) activity. (**F**) Representative hematoxylin and eosin (H&E) staining of myocardial tissue (scale bar = 50 µm). (**G**) Semi-quantitative histological injury scoring. (**H**) Masson’s trichrome staining (scale bar = 50 µm). (**I**) Quantification of fibrotic area. Data are expressed as mean ± SEM. * *p* < 0.05, ** *p* < 0.01, *** *p* < 0.001; ns, not significant.

Isoprenaline (ISO) administration induced pronounced myocardial injury, evidenced by significant elevations in serum cardiac troponin I (cTnI), creatine kinase-MB (CK-MB), and lactate dehydrogenase (LDH) in the model (MOD) group compared with controls (CON) (all *p* < 0.001; Figure 1A–C). Treatment with sodium butyrate (SB) significantly reduced cTnI (*p* < 0.05), CK-MB (*p* < 0.01), and LDH (*p* < 0.01), indicating attenuation of cardiomyocyte damage. ISO also triggered oxidative stress, reflected by increased myocardial malondialdehyde (MDA) and decreased superoxide dismutase (SOD) activity (*p* < 0.001 and *p* < 0.01, respectively; Figure 1D,E). SB significantly reduced MDA levels and restored SOD activity relative to untreated MOD rats (*p* < 0.05–0.01). Histological analysis confirmed severe myocardial necrosis, inflammatory infiltration, and myofibrillar disorganization in ISO-treated rats, which were markedly improved by SB (Figure 1F). Injury scoring demonstrated significant protection in the MOD-SB group (*p* < 0.01; Figure 1G). Masson’s trichrome staining further revealed extensive interstitial fibrosis in the MOD group that was substantially reduced by SB (*p* < 0.01; Figure 1H,I).

### 3.2. SB Protected Intestinal Barrier Structure in ISO-Induced Injury

**Figure 2:**
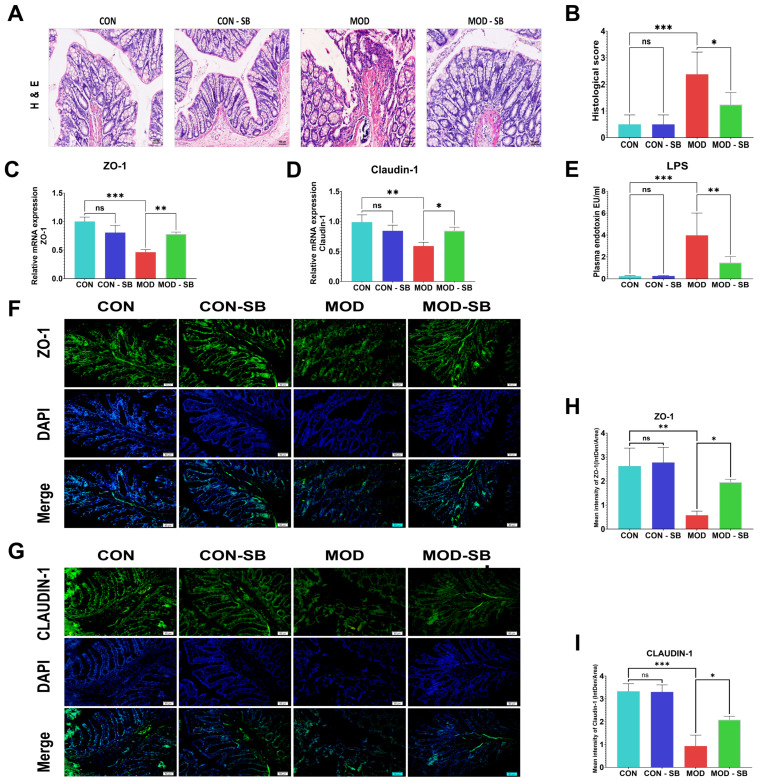
Sodium butyrate restored intestinal barrier structure and tight-junction integrity after ISO-induced myocardial injury. (**A**) Representative H&E staining of colonic tissue (scale bar = 100 µm). (**B**) Semi-quantitative histological injury scoring. Colonic mRNA expression of tight junction genes: (**C**) *Tjp1* (ZO-1), (**D**) *Cldn1* (claudin-1) determined by qRT-PCR. (**E**) Plasma endotoxin (lipopolysaccharide, LPS) levels. Representative immunofluorescence staining of tight junction proteins in colonic epithelium: (**F**) ZO-1, (**G**) claudin-1 (green fluorescence; nuclei counterstained with DAPI, blue) (scale bar = 50 µm). Quantification of fluorescence intensity for (**H**) ZO-1 and (**I**) claudin-1. Data are expressed as mean ± SEM. * *p* < 0.05, ** *p* < 0.01, *** *p* < 0.001; ns, not significant.

Colon histology showed severe epithelial disruption, crypt disorganization, and inflammatory infiltration in ISO-treated rats, which were significantly alleviated by SB (*p* < 0.05; Figure 2A,B). ISO-induced myocardial injury markedly downregulated colonic mRNA expression of *Tjp1* (ZO-1) and *Cldn1* (claudin-1) (*p* < 0.01–0.001; Figure 2C,D), whereas SB significantly restored their expression. Circulating endotoxin levels were elevated in the MOD group (*p* < 0.001), indicating impaired barrier permeability, and were significantly reduced by SB (*p* < 0.01; Figure 2E). Immunofluorescence further confirmed disrupted junctional localization of tight junction proteins in the ISO-induced myocardial injury model, while SB restored continuous epithelial distribution and fluorescence intensity for ZO-1 and claudin-1 (*p* < 0.05; Figure 2F–I).

### 3.3. SB Attenuated ISO-Induced Systemic Inflammation

**Figure 3:**
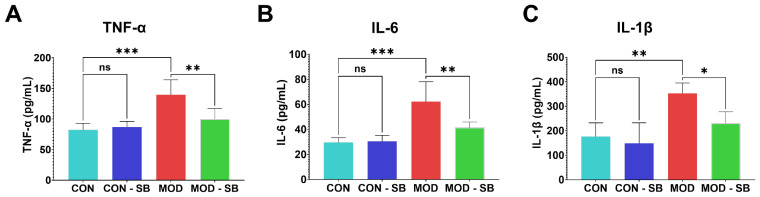
Sodium butyrate suppressed systemic inflammation induced by isoprenaline injury. Serum concentrations of pro-inflammatory cytokines: (**A**) tumor necrosis factor-alpha (TNF-α), (**B**) interleukin-6 (IL-6), and (**C**) interleukin-1β (IL-1β), measured by ELISA. Data are expressed as mean ± SEM. * *p* < 0.05, ** *p* < 0.01, *** *p* < 0.001; ns, not significant.

ISO-induced myocardial injury triggered a pronounced systemic inflammatory response, with significantly elevated serum TNF-α, IL-6, and IL-1β in the MOD group compared with CON (all *p* < 0.01–0.001; Figure 3A–C). SB treatment markedly reduced these cytokine levels (*p* < 0.05–0.01), confirming potent systemic anti-inflammatory activity.

### 3.4. SB Inhibited TLR4/NF-κB Signaling in ISO-Induced Cardiac Injury

**Figure 4:**
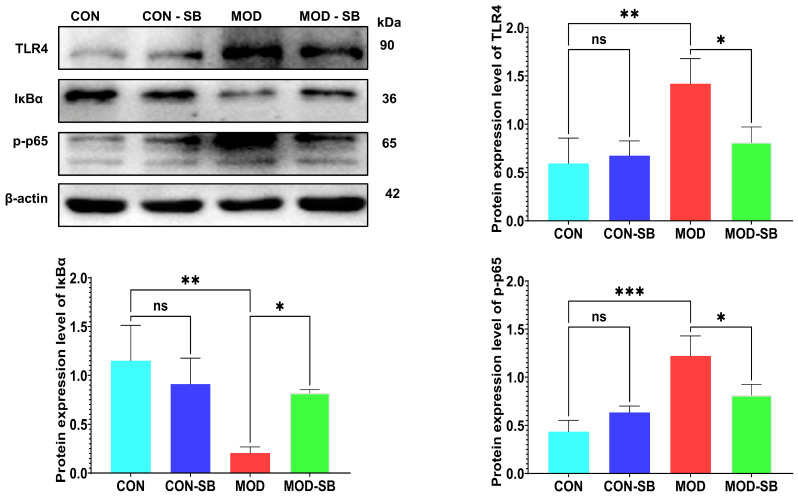
Sodium butyrate inhibited cardiac TLR4/NF-κB signaling activation after isoprenaline-induced injury. (**A**) Representative Western blot images showing protein expression of TLR4, IκBα, phosphorylated NF-κB p65 (p-p65), and β-actin (loading control) in cardiac tissue. Densitometric quantification of (**B**) TLR4, (**C**) IκBα, and (**D**) p-p65 protein expression, each normalized to the β-actin band from the same lane. Data are expressed as mean ± SEM. * *p* < 0.05, ** *p* < 0.01, *** *p* < 0.001; ns, not significant.

To elucidate the molecular mechanism underlying SB-mediated cardioprotection, the TLR4/NF-κB inflammatory signaling pathway was evaluated by Western blotting. ISO-induced myocardial injury markedly increased cardiac TLR4 expression and promoted IκBα degradation together with enhanced NF-κB p65 phosphorylation in the MOD group compared with CON (all *p* < 0.05; Figure 4A–D), indicating robust activation of pro-inflammatory signaling. SB administration significantly suppressed TLR4 upregulation, restored IκBα protein levels, and reduced NF-κB p65 phosphorylation relative to untreated MOD rats (all *p* < 0.05). These findings demonstrate that SB inhibited TLR4-dependent NF-κB inflammatory signaling, providing a mechanistic basis for its anti-inflammatory and cardioprotective effects.

### 3.5. SB Restored Gut Microbiota Imbalance After ISO-Induced Injury

**Figure 5:**
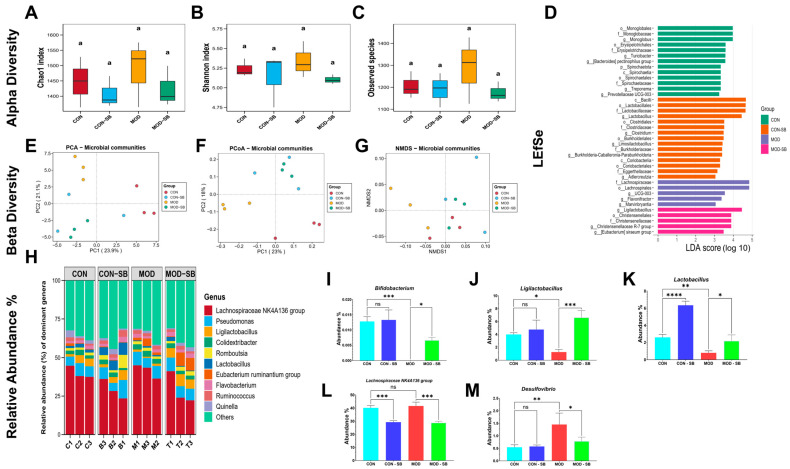
Sodium butyrate remodeled gut microbial community structure in isoprenaline-injured rats. Alpha diversity indices (Chao1, Shannon, observed species) (**A**–**C**); shared lowercase letter “a” above boxplots indicates groups not statistically different from one another (Kruskal–Wallis test, *p* > 0.05). LEfSe analysis showing discriminative taxa among groups (linear discriminant analysis score LDA ≥ 2.0, α = 0.05) (**D**). Beta diversity ordination by PCA, PCoA, and NMDS (**E**–**G**); sample labels C1-3, B1-3, M1-3, and T1-3 denote individual animals within the CON, CON-SB, MOD, and MOD-SB groups, respectively. Genus-level relative abundance composition (**H**) and relative abundance of selected genera significantly altered by ISO-induced myocardial injury and/or SB treatment (**I**–**M**). Data are expressed as mean ± SEM. * *p* < 0.05, ** *p* < 0.01, *** *p* < 0.001, **** *p* < 0.0001; ns, not significant.

Sequencing yielded 2082 OTUs after quality control (rarefied to 31,059 reads/sample). Alpha diversity indices (Chao1, Shannon, and observed species) showed no significant differences among groups (Figure 5A–C). While this pattern is consistent with some acute inflammatory models in which rapid compositional shifts may precede detectable changes in overall richness [28,29], alternative explanations should also be considered. These include the possibility that the four-week treatment duration was sufficient to alter community composition but not overall diversity. These considerations should be taken into account when interpreting the alpha diversity data. However, beta diversity analyses using principal component analysis (PCA; PC1 = 23.9%, PC2 = 21.1%), principal coordinate analysis (PCoA; PC1 = 23%, PC2 = 18%), and non-metric multidimensional scaling (NMDS; stress score = 0.06) demonstrated clear microbial community separation in the ISO-induced myocardial injury model that was partially restored by SB (Figure 5E–G). LEfSe analysis revealed distinct microbial signatures across experimental groups (Figure 5D). The control (CON) group was enriched in fiber-fermenting taxa such as *Monoglobus* and related orders (LDA = 3.9–4.0). The CON-SB group showed increased probiotic taxa, including *Lactobacillus*, *Lactobacillaceae*, and *Lactobacillales* (LDA = 4.7–4.9). In contrast, ISO-induced myocardial injury (MOD) rats displayed enrichment of pro-inflammatory genera, including UCG-003, *Flavonifractor*, and *Marvinbryantia* (LDA = 3.3–3.8), indicating dysbiosis. SB intervention in the MOD-SB group reversed these alterations, enriching beneficial commensals, including *Lactobacillus*, *Christensenellaceae*, and *Christensenellales* (LDA = 3.9–4.7).

At the phylum level, Pseudomonadota increased from CON to MOD (~8% to ~12%) with a partial reduction in MOD-SB (~10%). Bacteroidota remained relatively stable (~9–10%), whereas Actinomycetota and Thermodesulfobacteriota were enriched in MOD and remained modestly elevated in MOD-SB (Supplementary Figure S1). At the genus level, ISO-induced myocardial injury significantly depleted beneficial bacteria, including *Bifidobacterium*, *Ligilactobacillus*, and *Lactobacillus* (Figure 5I–K), while promoting the growth of *Lachnospiraceae NK4A136* group and the potentially pathogenic genus *Desulfovibrio* (Figure 5L,M). Specifically, SB significantly increased the relative abundance of *Bifidobacterium* (*p* < 0.05) and *Ligilactobacillus* (*p* < 0.001), while significantly decreasing *Desulfovibrio* (*p* < 0.05) and Lachnospiraceae NK4A136 group (*p* < 0.001), indicating a shift from a pro-inflammatory to an anti-inflammatory microbial community structure.

### 3.6. SB Reprogrammed Circulating Metabolites in ISO-Induced Myocardial Injury

**Figure 6:**
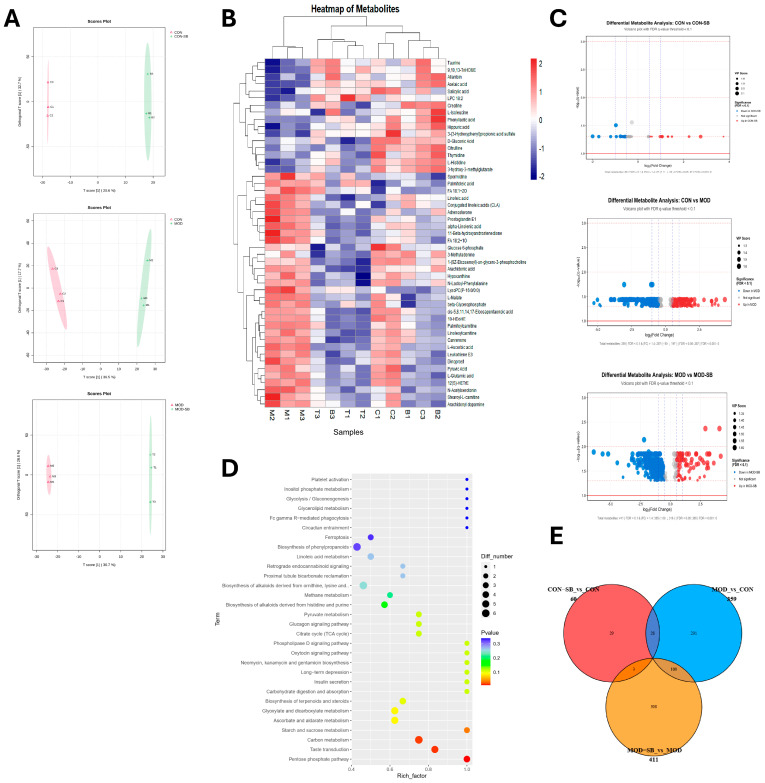
Serum metabolomic remodeling by sodium butyrate in isoprenaline-induced myocardial injury. (**A**) OPLS-DA score plots showing group separation across three pairwise comparisons; sample labels C1-3, B1-3, M1-3, and T1-3 denote individual animals within CON, CON-SB, MOD, and MOD-SB groups, respectively. (**B**) Heatmap of representative differential metabolites with hierarchical clustering. (**C**) Volcano plots of differential features for each pairwise comparison; sample labels C1-3, B1-3, M1-3, and T1-3 denote individual animals within the CON, CON-SB, MOD, and MOD-SB groups, respectively. (**D**) KEGG pathway enrichment analysis of SB-responsive metabolites. (**E**) Venn diagram summarizing overlaps of differential metabolites among key comparisons.

Untargeted metabolomics identified extensive ISO injury-induced metabolic disturbances that were markedly normalized by SB. A total of 1904 features in positive ionization mode and 655 in negative mode were initially detected. After QC filtering (RSD < 30%) and blank subtraction, 1487 (positive mode) and 493 (negative mode) high-quality features were retained. Orthogonal partial least squares–discriminant analysis (OPLS-DA) plots demonstrated a distinct metabolic signature in the MOD group that was clearly differentiated from the CON group (Figure 6A). SB treatment markedly restructured the dysregulated metabolome, shifting the profile of the MOD-SB group toward that of the CON group. Volcano plots further depicted the extent of this disruption, highlighting numerous significantly upregulated and downregulated metabolites in the MOD group compared to controls (Figure 6C). Of 359 metabolites dysregulated by ISO-induced myocardial injury (MOD group compared with CON), 100 were significantly reversed following SB treatment (MOD-SB group compared with MOD) (Figure 6E). The expression trends of several representative metabolites illustrate this remodeling, demonstrating how SB treatment frequently shifted their levels back toward the control state (Figure 6B). KEGG pathway enrichment analysis of the metabolites modulated by SB revealed significant alterations in pathways essential for cardiovascular health, including platelet activation, inositol phosphate metabolism, and glycolysis/gluconeogenesis (Figure 6D).

### 3.7. SB Coordinated Microbiome and Metabolome Normalization

**Figure 7:**
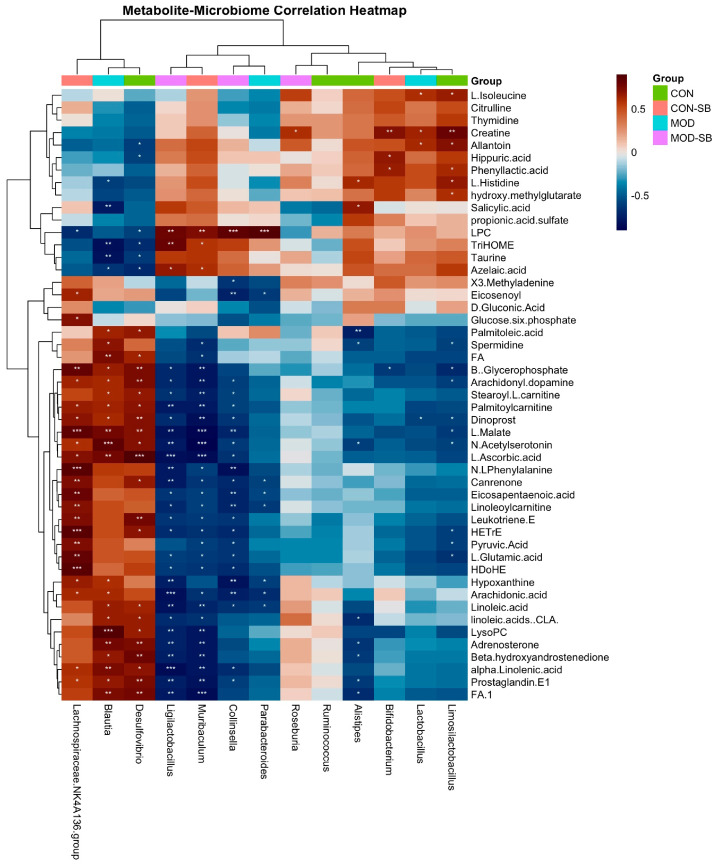
Microbiome–metabolome correlation heatmap shows SB-dependent reorganization of gut-systemic metabolic associations. Spearman correlation heatmap between differential bacterial genera and representative differential serum metabolites. Color intensity reflects Spearman’s correlation coefficient (ρ). Both genera and metabolites were hierarchically clustered to identify association modules. Asterisks denote correlations that remained significant after Benjamini–Hochberg false discovery rate correction (*p* < 0.05). * *p* < 0.05, ** *p* < 0.01, *** *p* < 0.001.

To link SB-induced microbiota shifts with circulating metabolic remodeling, we performed Spearman correlation analysis between differential genera and representative differential serum metabolites (selected from SB-responsive features). The correlation heatmap (Figure 7) revealed two opposing association modules. SB-enriched genera, including *Ligilactobacillus* and *Bifidobacterium*, tended to correlate positively with features related to energy and redox homeostasis. In contrast, ISO-enriched genera, including *Desulfovibrio* and *Lachnospiraceae NK4A136* group, showed the opposite pattern and correlated positively with several lipid-related features, including putatively annotated lipid mediator-associated signals. Overall, these clustered correlation patterns support coordinated microbiome–metabolome remodeling following SB treatment and are consistent with the restoration of gut–heart axis homeostasis.

## 4. Discussion

Previous investigations have demonstrated the cardioprotective properties of butyrate mediated through neural regulation, epigenetic modulation, and immune reprogramming, including macrophage polarization [30,31,32,33]. However, the upstream contribution of the intestinal ecosystem and its role in transmitting cardioprotective signals to the host have remained insufficiently defined. The present study addressed this gap by providing integrated histological, molecular, microbiome, and metabolomic evidence showing that sodium butyrate (SB) confers cardioprotection primarily through the preservation of gut barrier integrity, suppression of TLR4/NF-κB driven inflammation, and restoration of microbiota-dependent metabolic homeostasis.

Consistent with the established literature, the isoprenaline-induced myocardial injury model reproduced the key pathological features of myocardial injury, including cardiomyocyte necrosis, oxidative stress, inflammation, and interstitial fibrosis [34]. SB markedly reduced circulating cardiac injury markers, improved myocardial architecture, and attenuated fibrotic remodeling, indicating robust structural and biochemical cardioprotection. These benefits were accompanied by the significant suppression of systemic pro-inflammatory cytokines (TNF-α, IL-6, and IL-1β), supporting the well-recognized anti-inflammatory capacity of butyrate [35].

Mechanistically, our Western blot data demonstrate that SB inhibited TLR4 activation, prevented IκBα degradation, and reduced NF-κB p65 phosphorylation, thereby identifying TLR4/NF-κB suppression as a central molecular mechanism underlying the observed anti-inflammatory and cardioprotective effects [15,36]. Given that excessive NF-κB activation is a key determinant of post-MI remodeling and heart failure progression [37,38], this pathway provides a mechanistic bridge between intestinal regulation and myocardial protection. Critically, the suppression of TLR4/NF-κB signaling is not an isolated cardiac event but rather the downstream consequence of upstream gut barrier restoration. ISO-induced myocardial injury caused marked colonic inflammation, disruption of epithelial architecture, and downregulation of tight junction proteins (ZO-1, claudin-1), which were accompanied by elevated circulating endotoxin levels, indicative of barrier failure. These findings align with the concept of a “cardio-intestinal syndrome,” in which cardiac dysfunction leads to intestinal hypoperfusion, mucosal inflammation, and microbial translocation that further amplifies systemic inflammation and myocardial injury [15,39,40,41,42].

SB effectively restored tight junction expression, normalized endotoxin levels, and improved intestinal histology, consistent with the known role of butyrate as the principal energy substrate for colonocytes and a direct enhancer of epithelial barrier function [43,44]. By “sealing the gut,” SB eliminates a major source of pathogen-associated molecular patterns (PAMPs), particularly lipopolysaccharide (LPS), which are the primary danger signals that trigger myocardial TLR4 activation. Thus, gut barrier preservation emerges as a pivotal upstream mechanism limiting inflammatory amplification after ISO-induced myocardial injury. This establishes a mechanistic cascade wherein gut barrier restoration reduces endotoxemia, which in turn suppresses TLR4/NF-κB signaling, ultimately leading to attenuated myocardial inflammation and injury [16,45,46].

Microbiome profiling provided systems-level insight into this protective process. ISO-induced myocardial injury altered the community structure while maintaining diversity indices, consistent with acute inflammatory stress patterns [28,29]. ISO-induced myocardial injury depleted beneficial commensals such as *Bifidobacterium* and *Lactobacillus* while enriching potentially pro-inflammatory taxa, a dysbiotic configuration previously associated with cardiovascular pathology [47,48]. SB reversed these shifts, promoting probiotic and SCFA-associated genera and suppressing detrimental microbes. The restoration of *Lactobacillus*-dominated ecological balance is particularly relevant given its documented anti-inflammatory, metabolic, and cardioprotective functions [49,50], suggesting that SB initiates a positive microbiota-mediated feedback loop that reinforces intestinal and systemic homeostasis.

Serum metabolomics further demonstrated that microbial remodeling translated into functional metabolic reprogramming. Myocardial injury induced widespread metabolic disruption involving pathways linked to mitochondrial dysfunction, lipid metabolism, and platelet activation. Among the key metabolites reversed by SB treatment, palmitoylcarnitine, a long-chain acylcarnitine that accumulated in the MOD group, was partially normalized by SB, reflecting the restoration of mitochondrial long-chain fatty acid β-oxidation capacity, which is characteristically impaired in cardiac energetic failure [51,52,53]. Hippuric acid, a gut-microbiota-derived conjugation product of phenylalanine catabolism, was reduced in the MOD group and restored by SB treatment, consistent with the recovery of microbial aromatic amino acid metabolism and previously reported associations between hippurate and gut microbiome diversity [54,55]. SB normalized a substantial subset of these metabolites and enriched the pathways associated with energy metabolism and cardiovascular protection [22,56].

Correlation analysis revealed coordinated statistical associations between beneficial bacteria and cardioprotective metabolites, whereas dysbiosis-associated taxa were associated with potentially adverse metabolic signatures; these findings are correlational in nature and do not establish direct causality. Notably, *Desulfovibrio* has been linked in prior studies to a pro-inflammatory gut environment and adverse cardiometabolic phenotypes; its reduction by SB may therefore contribute to a less inflammatory gut–heart axis milieu [57,58,59]. The ability of SB to normalize these microbial and metabolic disturbances reinforces the functional integration of the gut–heart axis and supports its systemic metabolic benefits [54,55]. These findings are consistent with functional host–microbe metabolic integration and support the gut–heart axis as a viable therapeutic target; however, whether microbial shifts directly drive the observed metabolic changes and cardiac protection requires causal validation, for example, through fecal microbiota transplantation experiments.

The microbiome–metabolome correlation heatmap (Figure 7) further supports coordinated SB-associated remodeling: SB-enriched genera clustered with features linked to energy/redox homeostasis, whereas ISO-enriched genera clustered with lipid-related features. Although correlation does not establish causality, these structured association modules align with the observed improvements in intestinal barrier integrity, reduced endotoxemia, and suppression of cardiac TLR4/NF-κB activation, providing convergent evidence for gut–heart axis involvement in SB cardioprotection.

This study employed an integrated multi-omics approach combining histopathology, molecular signaling analysis, 16S rRNA sequencing, and untargeted metabolomics to systematically interrogate the gut–heart axis at multiple biological scales. The isoprenaline model, while not replicating coronary occlusion–reperfusion injury, offers distinct advantages for mechanistic studies. It reliably induces diffuse myocardial damage with high reproducibility, minimal surgical mortality, and permits the isolated evaluation of gut-mediated cardioprotection without ischemia–reperfusion confounders [34,60]. This standardized injury model enabled the clear demonstration of intestinal barrier disruption and TLR4/NF-κB activation as critical pathophysiological nodes amenable to therapeutic intervention. It is important to note, however, that the isoprenaline model generates catecholamine-mediated diffuse myocardial necrosis through adrenergic overstimulation, which is mechanistically distinct from coronary occlusion–reperfusion injury in which regional ischemia, neutrophil-dominated early inflammation, and microvascular obstruction play central roles. Therefore, while the LPS–TLR4/NF-κB axis is clearly activated in the present model, likely driven by sympathetically mediated intestinal hypoperfusion and barrier failure, its relative quantitative contribution to cardiac injury may differ in ischemic infarction settings. Caution should therefore be exercised when extrapolating these mechanistic conclusions to clinical myocardial infarction, and validation in coronary ligation or ischemia–reperfusion models is warranted to establish the generalizability of these findings.

The correlative nature of microbiome–metabolome associations represents a limitation inherent to observational multi-omics studies; accordingly, the directional relationships observed between beneficial taxa (*Lactobacillus*, *Bifidobacterium*), normalized metabolites, and attenuated cardiac injury should be interpreted as convergent associative evidence rather than mechanistic proof of host–microbe integration. Causal validation through fecal microbiota transplantation is required to confirm that microbial shifts are directly responsible for the observed cardioprotective outcomes. An additional limitation is the exclusive use of male Sprague-Dawley rats, which precludes assessment of sex-dependent differences in gut microbiota composition, intestinal barrier function, and inflammatory responsiveness. Emerging evidence indicates that gut microbiota composition and SCFA metabolism differ substantially between sexes and may modulate cardiovascular inflammatory responses differently [61]. Future studies incorporating female animals are therefore warranted to determine whether the cardioprotective effects of sodium butyrate through the gut–heart axis are applicable across sexes.

Despite these considerations, the multi-level convergence of findings spanning cardiac injury markers, inflammatory signaling, intestinal barrier function, microbial composition, and circulating metabolites provides robust mechanistic evidence positioning the gut–heart axis as a viable therapeutic target and sodium butyrate as a promising adjunctive intervention for myocardial infarction.

## 5. Conclusions

In conclusion, this study demonstrates that sodium butyrate (SB) conferred cardioprotection in an isoprenaline-induced myocardial injury model through a coordinated, multi-level restoration of the gut–heart axis. SB significantly attenuated cardiomyocyte necrosis, oxidative stress, and interstitial fibrosis, as evidenced by reduced serum cTnI, CK-MB, and LDH levels and improved histopathological findings. Mechanistically, SB preserved intestinal epithelial barrier integrity by upregulating the expression of tight junction proteins ZO-1 and claudin-1, thereby reducing circulating endotoxin levels and subsequent activation of the cardiac TLR4/NF-κB inflammatory signaling pathway. At the microbial level, SB shifted the gut community from a pro-inflammatory dysbiotic state—characterized by the enrichment of *Desulfovibrio* and *Lachnospiraceae NK4A136* group—toward a beneficial composition enriched in *Ligilactobacillus* and *Bifidobacterium*. Untargeted serum metabolomics further revealed that SB normalized a substantial proportion of ISO-induced metabolic disturbances, particularly those involving energy metabolism, lipid handling, and platelet-related pathways. The microbiome–metabolome correlation analysis supported the coordinated remodeling of the gut–systemic metabolic interface following SB treatment. Collectively, these findings position SB as a promising gut-targeted cardioprotective intervention, with the gut–heart axis—specifically the gut barrier–endotoxemia–TLR4/NF-κB cascade—as the central therapeutic node.

Future causal validation through fecal microbiota transplantation and sex-inclusive experimental designs will be essential to generalize these findings.

## Figures and Tables

**Table 1:** Primer sequences used for qRT-PCR.

Gene Name	Forward Sequence (5′ → 3′)	Reverse Sequence (5′ → 3′)
*Tjp1* (ZO-1)	GTGCTCACCAGGGTCAAAAT	GGCTTAAAGCTGGCAGTGTC
*Cldn1* (Claudin-1)	GTCCCCGGAAAACAACCTCT	CAGCCAAGACCCTCATAGCC
*Actb* (β-actin)	CATGTACGTTGCTATCCAGGC	CAGGGTACATGGTGGTGAC

## Data Availability

The 16S rRNA sequencing data were deposited in the NCBI Sequence Read Archive under accession PRJNA1358548.

## Supplementary Materials

The following supporting information can be downloaded at: https://www.mdpi.com/article/10.3390/cimb48050501/s1.

## References

[1] Wang Y., Li J., Bilal H., Yu X., Sun L. (2025). The Rising Tide of Coronary Crisis: Decoding Age-Specific Disparities in Ischemic Heart Disease Burden Through the Global Burden of Disease Study 2021 Revelations: An Ecological Study. Health Sci. Rep..

[2] Heusch G. (2024). Myocardial ischemia/reperfusion: Translational pathophysiology of ischemic heart disease. Med.

[3] Francisco J., Del Re D.P. (2023). Inflammation in Myocardial Ischemia/Reperfusion Injury: Underlying Mechanisms and Therapeutic Potential. Antioxidants.

[4] González-Montero J., Brito R., Gajardo A.I., Rodrigo R. (2018). Myocardial reperfusion injury and oxidative stress: Therapeutic opportunities. World J. Cardiol..

[5] Kibel A., Lukinac A.M., Dambic V., Juric I., Selthofer-Relatic K. (2020). Oxidative Stress in Ischemic Heart Disease. Oxidative Med. Cell. Longev..

[6] Gao S., Huang S., Liu X., Yu M., Li W. (2025). Significance of Residual Inflammatory Risk and Persistent Inflammation in Patients with Myocardial Infarction with Nonobstructive Coronary Arteries. J. Inflamm. Res..

[7] Volpe M., Presta V. (2018). Inflammatory residual risk: An emerging target to reduce cardiovascular disease?. Clin. Cardiol..

[8] Mylavarapu M., Tiwari A., Kaur H., Vempati R., Kumar H., Kodali L.S.M., Khan K.G., Dadana S., Garcia I., Cabrera F.E.P. (2026). The Gut-Heart Axis: A Comprehensive Review of Microbiota’s Role in Cardiovascular Health and Disease and Emerging Therapeutic Strategies. Cardiol. Res. Pract..

[9] Abdulrahim A.O., Doddapaneni N.S.P., Salman N., Giridharan A., Thomas J., Sharma K., Abboud E., Rochill K., Shreelakshmi B., Gupta V. (2025). The gut-heart axis: A review of gut microbiota, dysbiosis, and cardiovascular disease development. Ann. Med. Surg..

[10] Nireeksha, Maniangat Luke A., Kumari N.S., Hegde M.N., Hegde N.N. (2025). Metabolic interplay of SCFA’s in the gut and oral microbiome: A link to health and disease. Front. Oral Health.

[11] Caffaratti C., Plazy C., Mery G., Tidjani A.R., Fiorini F., Thiroux S., Toussaint B., Hannani D., Le Gouellec A. (2021). What We Know So Far about the Metabolite-Mediated Microbiota-Intestinal Immunity Dialogue and How to Hear the Sound of This Crosstalk. Metabolites.

[12] Mann E.R., Lam Y.K., Uhlig H.H. (2024). Short-chain fatty acids: Linking diet, the microbiome and immunity. Nat. Rev. Immunol..

[13] Zhou X., Li J., Guo J., Geng B., Ji W., Zhao Q., Li J., Liu X., Liu J., Guo Z. (2018). Gut-dependent microbial translocation induces inflammation and cardiovascular events after ST-elevation myocardial infarction. Microbiome.

[14] Dmytriv T.R., Storey K.B., Lushchak V.I. (2024). Intestinal barrier permeability: The influence of gut microbiota, nutrition, and exercise. Front. Physiol..

[15] Zhang C., Teng X., Cao Q., Deng Y., Yang M., Wang L., Rui D., Ling X., Wei C., Chen Y. (2025). Gut microbiota dysbiosis exacerbates heart failure by the LPS-TLR4/NF-κB signalling axis: Mechanistic insights and therapeutic potential of TLR4 inhibition. J. Transl. Med..

[16] Zhao J., Zhang Q., Cheng W., Dai Q., Wei Z., Guo M., Chen F., Qiao S., Hu J., Wang J. (2023). Heart-gut microbiota communication determines the severity of cardiac injury after myocardial ischaemia/reperfusion. Cardiovasc. Res..

[17] Fusco W., Lorenzo M.B., Cintoni M., Porcari S., Rinninella E., Kaitsas F., Lener E., Mele M.C., Gasbarrini A., Collado M.C. (2023). Short-Chain Fatty-Acid-Producing Bacteria: Key Components of the Human Gut Microbiota. Nutrients.

[18] Deleu S., Machiels K., Raes J., Verbeke K., Vermeire S. (2021). Short chain fatty acids and its producing organisms: An overlooked therapy for IBD?. eBioMedicine.

[19] Yan H., Ajuwon K.M. (2017). Butyrate modifies intestinal barrier function in IPEC-J2 cells through a selective upregulation of tight junction proteins and activation of the Akt signaling pathway. PLoS ONE.

[20] Siddiqui M.T., Cresci G.A.M. (2021). The Immunomodulatory Functions of Butyrate. J. Inflamm. Res..

[21] Wang J., Zhao Q., Zhang S., Liu J., Fan X., Han B., Hou Y., Ai X. (2026). Microbial short chain fatty acids: Effective histone deacetylase inhibitors in immune regulation (Review). Int. J. Mol. Med..

[22] Chen H.C., Liu Y.W., Chang K.C., Wu Y.W., Chen Y.M., Chao Y.K., You M.Y., Lundy D.J., Lin C.J., Hsieh M.L. (2023). Gut butyrate-producers confer post-infarction cardiac protection. Nat. Commun..

[23] Markowiak-Kopeć P., Śliżewska K. (2020). The Effect of Probiotics on the Production of Short-Chain Fatty Acids by Human Intestinal Microbiome. Nutrients.

[24] Nagpal R., Wang S., Ahmadi S., Hayes J., Gagliano J., Subashchandrabose S., Kitzman D.W., Becton T., Read R., Yadav H. (2018). Human-origin probiotic cocktail increases short-chain fatty acid production via modulation of mice and human gut microbiome. Sci. Rep..

[25] Hosseini A., Rajabian A., Sobhanifar M.A., Alavi M.S., Taghipour Z., Hasanpour M., Iranshahi M., Boroumand-Noughabi S., Banach M., Sahebkar A. (2022). Attenuation of isoprenaline-induced myocardial infarction by Rheum turkestanicum. Biomed. Pharmacother..

[26] Zhang L., Deng M., Lu A., Chen Y., Chen Y., Wu C., Tan Z., Boini K.M., Yang T., Zhu Q. (2019). Sodium butyrate attenuates angiotensin II-induced cardiac hypertrophy by inhibiting COX2/PGE2 pathway via a HDAC5/HDAC6-dependent mechanism. J. Cell. Mol. Med..

[27] Yu Z., Han J., Chen H., Wang Y., Zhou L., Wang M., Zhang R., Jin X., Zhang G., Wang C. (2021). Oral Supplementation with Butyrate Improves Myocardial Ischemia/Reperfusion Injury via a Gut-Brain Neural Circuit. Front. Cardiovasc. Med..

[28] Dong C., Yang Y., Wang Y., Hu X., Wang Q., Gao F., Sun S., Liu Q., Li L., Liu J. (2023). Gut microbiota combined with metabolites reveals unique features of acute myocardial infarction patients different from stable coronary artery disease. J. Adv. Res..

[29] Zheng A., Yi H., Li F., Han L., Yu J., Cheng X., Su H., Hong K., Li J. (2019). Changes in Gut Microbiome Structure and Function of Rats with Isoproterenol-Induced Heart Failure. Int. Heart J..

[30] Jiang X., Huang X., Tong Y., Gao H. (2020). Butyrate improves cardiac function and sympathetic neural remodeling following myocardial infarction in rats. Can. J. Physiol. Pharmacol..

[31] Song T., Guan X., Wang X., Qu S., Zhang S., Hui W., Men L., Chen X. (2021). Dynamic modulation of gut microbiota improves post-myocardial infarct tissue repair in rats via butyric acid-mediated histone deacetylase inhibition. FASEB J..

[32] Qian X., Liu A., Liang C., He L., Xu Z., Tang S. (2022). Analysis of gut microbiota in patients with acute myocardial infarction by 16S rRNA sequencing. Ann. Transl. Med..

[33] Wang X., Dong Y., Huang R., Wang F., Xie J., Liu H., Wang Y., Wang Y., Luo S., Hu D. (2024). The Role of Short-Chain Fatty Acids in Myocardial Ischemia-Reperfusion Injury. Curr. Nutr. Rep..

[34] Bader Eddin L., Nagoor Meeran M.F., Kumar Jha N., Goyal S.N., Ojha S. (2025). Isoproterenol mechanisms in inducing myocardial fibrosis and its application as an experimental model for the evaluation of therapeutic potential of phytochemicals and pharmaceuticals. Anim. Models Exp. Med..

[35] Segain J.P., Raingeard de la Blétière D., Bourreille A., Leray V., Gervois N., Rosales C., Ferrier L., Bonnet C., Blottière H.M., Galmiche J.P. (2000). Butyrate inhibits inflammatory responses through NFkappaB inhibition: Implications for Crohn’s disease. Gut.

[36] Chong A.J., Shimamoto A., Hampton C.R., Takayama H., Spring D.J., Rothnie C.L., Yada M., Pohlman T.H., Verrier E.D. (2004). Toll-like receptor 4 mediates ischemia/reperfusion injury of the heart. J. Thorac. Cardiovasc. Surg..

[37] Hamid T., Guo S.Z., Kingery J.R., Xiang X., Dawn B., Prabhu S.D. (2011). Cardiomyocyte NF-κB p65 promotes adverse remodelling, apoptosis, and endoplasmic reticulum stress in heart failure. Cardiovasc. Res..

[38] Gordon J.W., Shaw J.A., Kirshenbaum L.A. (2011). Multiple facets of NF-κB in the heart: To be or not to NF-κB. Circ. Res..

[39] Zhou W., Cheng Y., Zhu P., Nasser M.I., Zhang X., Zhao M. (2020). Implication of Gut Microbiota in Cardiovascular Diseases. Oxidative Med. Cell. Longev..

[40] Sandek A., Anker S.D., von Haehling S. (2009). The gut and intestinal bacteria in chronic heart failure. Curr. Drug Metab..

[41] Snelson M., R Muralitharan R., Liu C.F., Markó L., Forslund S.K., Marques F.Z., Tang W.H.W. (2025). Gut-Heart Axis: The Role of Gut Microbiota and Metabolites in Heart Failure. Circ. Res..

[42] Matsiras D., Bezati S., Ventoulis I., Verras C., Parissis J., Polyzogopoulou E. (2023). Gut Failure: A Review of the Pathophysiology and Therapeutic Potentials in the Gut-Heart Axis. J. Clin. Med..

[43] Li X., Li R., You N., Zhao X., Li J., Jiang W. (2022). Butyric Acid Ameliorates Myocardial Fibrosis by Regulating M1/M2 Polarization of Macrophages and Promoting Recovery of Mitochondrial Function. Front. Nutr..

[44] Saban Güler M., Arslan S., Ağagündüz D., Cerqua I., Pagano E., Berni Canani R., Capasso R. (2025). Butyrate: A potential mediator of obesity and microbiome via different mechanisms of actions. Food Res. Int..

[45] Weiss S.L., Zhang D., Farooqi S., Wallace D.C. (2022). Sodium butyrate reverses lipopolysaccharide-induced mitochondrial dysfunction in lymphoblasts. J. Cell. Mol. Med..

[46] Yu J., Zhou L., Li G., Chen Z., Mudabbar M.S., Li L., Tang X., Jiang M., Zhang G., Liu X. (2025). Targeting gut-immune-heart modulate cardiac remodeling after acute myocardial infarction. Life Sci..

[47] Bocchio F., Mancabelli L., Milani C., Lugli G.A., Tarracchini C., Longhi G., De Conto F., Turroni F., Ventura M. (2025). Compendium of Bifidobacterium-based probiotics: Characteristics and therapeutic impact on human diseases. Microbiome Res. Rep..

[48] Centner A.M., Khalili L., Ukhanov V., Kadyan S., Nagpal R., Salazar G. (2023). The Role of Phytochemicals and Gut Microbiome in Atherosclerosis in Preclinical Mouse Models. Nutrients.

[49] Dicks L.M.T. (2025). Butyrate Produced by Gut Microbiota Regulates Atherosclerosis: A Narrative Review of the Latest Findings. Int. J. Mol. Sci..

[50] Shi L., Duan Y., Fang N., Zhang N., Yan S., Wang K., Hou T., Wang Z., Jiang X., Gao Q. (2025). Lactobacillus gasseri prevents ibrutinib-associated atrial fibrillation through butyrate. Europace.

[51] Wiggers H. (2025). Fatty acids in heart failure patients: Friend or foe?. Eur. Heart J..

[52] Hunter W.G., Kelly J.P., McGarrah R.W., Kraus W.E., Shah S.H. (2016). Metabolic Dysfunction in Heart Failure: Diagnostic, Prognostic, and Pathophysiologic Insights from Metabolomic Profiling. Curr. Heart Fail. Rep..

[53] Kim H.I., Raffler J., Lu W., Lee J.J., Abbey D., Saleheen D., Rabinowitz J.D., Bennett M.J., Hand N.J., Brown C. (2017). Fine Mapping and Functional Analysis Reveal a Role of SLC22A1 in Acylcarnitine Transport. Am. J. Hum. Genet..

[54] Pallister T., Jackson M.A., Martin T.C., Zierer J., Jennings A., Mohney R.P., MacGregor A., Steves C.J., Cassidy A., Spector T.D. (2017). Hippurate as a metabolomic marker of gut microbiome diversity: Modulation by diet and relationship to metabolic syndrome. Sci. Rep..

[55] He H., Xu H., Xu J., Zhao H., Lin Q., Zhou Y., Nie Y. (2020). Sodium Butyrate Ameliorates Gut Microbiota Dysbiosis in Lupus-Like Mice. Front. Nutr..

[56] Lu Y., Zhang Y., Zhao X., Shang C., Xiang M., Li L., Cui X. (2022). Microbiota-derived short-chain fatty acids: Implications for cardiovascular and metabolic disease. Front. Cardiovasc. Med..

[57] Murros K.E., Huynh V.A., Takala T.M., Saris P.E.J. (2021). Desulfovibrio Bacteria Are Associated with Parkinson’s Disease. Front. Cell. Infect. Microbiol..

[58] Mazidi M., Shekoohi N., Covic A., Mikhailidis D.P., Banach M. (2020). Adverse Impact of Desulfovibrio spp. and Beneficial Role of Anaerostipes spp. on Renal Function: Insights from a Mendelian Randomization Analysis. Nutrients.

[59] Yu J., Yang Y.N., Chen W., Hu J., Jin Z., Wu C., Li Y. (2025). Role of gut microbiota and derived metabolites in cardiovascular diseases. iScience.

[60] Flori L., Lazzarini G., Spezzini J., Pirone A., Calderone V., Testai L., Miragliotta V. (2024). The isoproterenol-induced myocardial fibrosis: A biochemical and histological investigation. Biomed. Pharmacother..

[61] Org E., Mehrabian M., Parks B.W., Shipkova P., Liu X., Drake T.A., Lusis A.J. (2016). Sex differences and hormonal effects on gut microbiota composition in mice. Gut Microbes.

