# Supplementary material for: Sodium Butyrate Attenuates Isoprenaline-Induced Myocardial Injury via Restoring the Gut–Heart Axis and Suppressing TLR4/NF-κB Signaling
Source: Curr Issues Mol Biol. 2026 May 13;48(5):501. doi: 10.3390/cimb48050501 (PMC13205053; doi:10.3390/cimb48050501)
Supplement: Supplementary file 1 [file cimb-48-00501-s001.zip › cimb-4301189-supplementary.pdf]

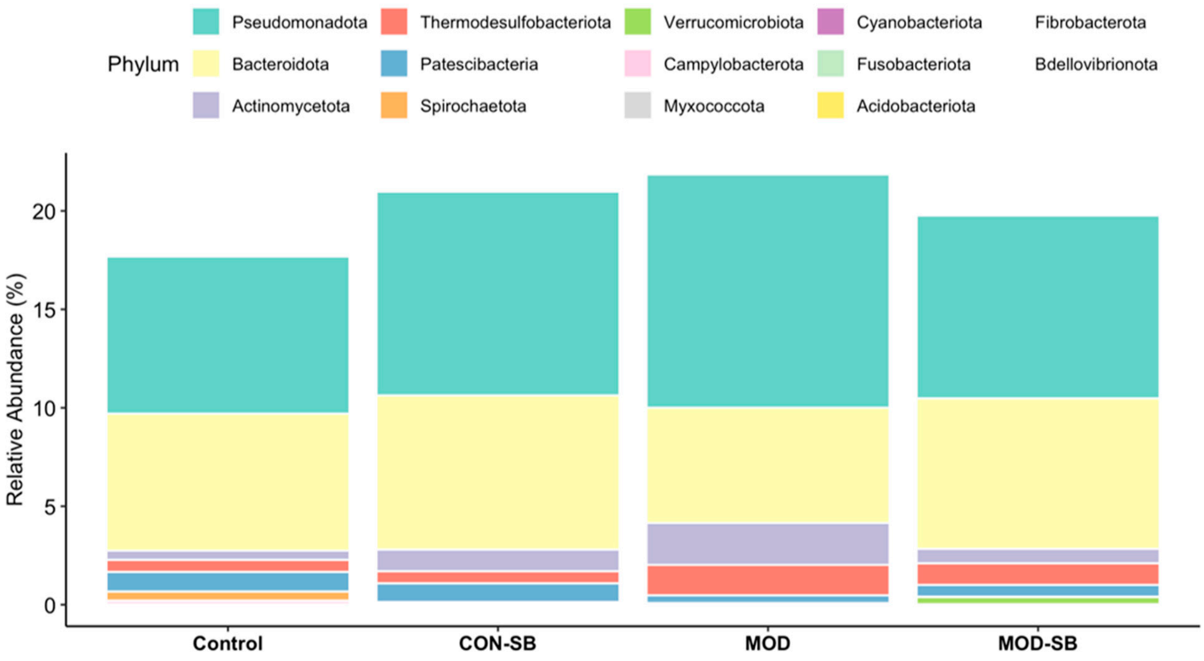

Supplementary Figure S1. Phylum-level relative abundance of gut microbiota across experimental groups. Stacked bar charts showing the relative abundance (%) of dominant bacterial phyla in fecal samples from Control, CON-SB, MOD, and MOD-SB groups.
